# Effect of Pre-Exposure to Chlorine Dioxide on the Susceptibility of Fecal Coliforms to Antibiotics

**DOI:** 10.3390/antibiotics11020215

**Published:** 2022-02-08

**Authors:** Joycelyn Quansah, Himabindu Gazula, Da Liu, Jinru Chen

**Affiliations:** 1 Department of Food Science and Technology, The University of Georgia, Griffin, GA 30223-1797, USA; joyquansah@ug.edu.gh (J.Q.); bindu.gazula@gmail.com (H.G.); liuda6280@gmail.com (D.L.); 2 Department of Nutrition and Food Science, University of Ghana, Legon P.O. Box LG 134, Ghana

**Keywords:** fecal coliforms, preadaptation, chlorine dioxide, sanitizer, antibiotic resistance

## Abstract

Adaptive exposure to sub-lethal concentrations of sanitizers was previously reported to offer cross-protection to bacteria against antibiotics. This study was undertaken to determine whether the pre-exposure of fecal coliforms to suboptimal concentrations of a chemical sanitizer, chlorine dioxide (ClO_2_), alters their susceptibility to certain antibiotics. Fecal coliforms isolated from fresh fruit packing facilities (n = 12) were adapted in ½ or ¼ of the manufacturer-recommended concentration of ClO_2_. The susceptibility of the adapted and non-adapted cells to 13 different antibiotics was determined by observing the changes in their minimal inhibitory concentrations (MICs). The results showed that preadaptation to the suboptimal concentrations of ClO_2_, in general, either decreased or did not change the MICs of the antibiotics against selected fecal coliform isolates, with only two exceptions; preadaptation increased the MICs of kanamycin against two of the fecal coliform isolates, and of nalidixic acid against one of the fecal coliform isolates. The results suggest that the use of ClO_2_ has a relatively low risk of inducing the resistance of fecal coliforms to antibiotics.

## 1. Introduction

Chemical sanitizers are widely used in food production environments for the decontamination of food contact surfaces and the prevention of microbial contamination [1,2]. Russell [3] stated that frequent use of sanitizers may impose selective pressure and contribute to the emergence of resistant microorganisms in food environments. Each sanitizer used by food processors has the manufacturer’s recommended working concentration and treatment protocols [4]; for example, the use of 5 parts per million (ppm) of chlorine dioxide (ClO_2_) solution is recommended to disinfect hard, non-porous food contact surfaces [5]. The application of sanitizers at concentrations lower than those recommended may allow some bacterial cells to evade routine sanitization treatment [6]. Repeated exposure to sub-inhibitory concentrations of sanitizers has been shown to result in enhanced tolerance of some bacterial species to chloramphenicol, erythromycin, and ciprofloxacin [7,8,9]. *Escherichia coli* cells exposed to sub-inhibitory concentrations of benzalkonium chloride had elevated tolerance to chloramphenicol. The cells of *Salmonella typhimurium* that were pre-exposed to sub-inhibitory concentrations of acidified sodium chlorite had increased resistance to streptomycin, erythromycin, rifampicin, and chloramphenicol [10].

At present, a few reports have demonstrated cross-protection between clinically used antibiotics and ClO_2_, a chemical sanitizer commonly used by the food industry. Pre-exposure to the sanitizer rendered the cells of *S. typhimurium* able to tolerate an increased concentration of antibiotics [1]. However, opposite findings were reported in several other studies [10,11,12,13]. Pre-exposure to ClO_2_ either decreased or did not alter the susceptibility of *Salmonella*, *E. coli*, and *Campylobacter* cells to several antibiotics frequently used in human medicine. The objective of this study was to determine whether pre-exposure to suboptimal concentrations of ClO_2_ alters the susceptibility of selected fecal coliforms to the antibiotics used in human medicine.

## 2. Results

**Figure 1:**
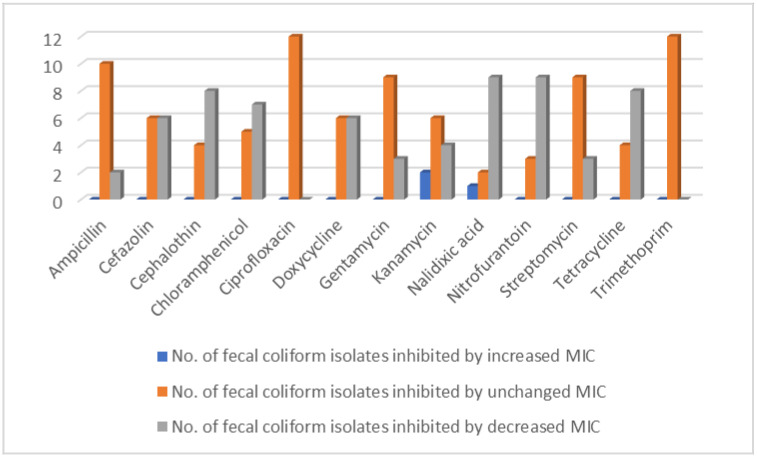
The number of tested fecal coliform isolates completely inhibited by a changed or unchanged minimal inhibitory concentration of antibiotics.

The minimum inhibitory concentrations (MICs) of selected antibiotics against fecal coliforms with or without preadaptation in suboptimal concentrations of ClO_2_ are shown in Table 1. The results suggest that the preadaptation process did not change the MICs of ciprofloxacin and trimethoprim against all twelve fecal coliform isolates used in the study (Figure 1).

A decrease in the MICs of cephalothin and tetracycline was observed with eight of the fecal coliform isolates, whilst the MICs against the other four fecal coliform isolates remained unchanged. A decrease in the MICs of gentamycin and streptomycin was observed with three of the fecal coliform isolates, and of cefazolin and doxycycline with six of the isolates, respectively, whilst the MICs remained the same for the rest of the fecal coliform isolates used in the study. The other three antibiotics, ampicillin, chloramphenicol, and nitrofurantoin, each had a decreased MIC against two, seven, and nine of the isolates used in this study, respectively. However, preadaptation with sub-lethal concentrations of ClO_2_ increased the MICs of kanamycin against two of the fecal coliform isolates, and decreased the MICs against four of the fecal coliform isolates. The MIC of nalidixic acid increased for one of the fecal coliform isolates and decreased for nine of the fecal coliform isolates.

The susceptibility of selected fecal coliforms to various antibiotics, as affected by preadaptation in suboptimal concentrations of ClO_2_, is shown in Table 2.

**Figure 2:**
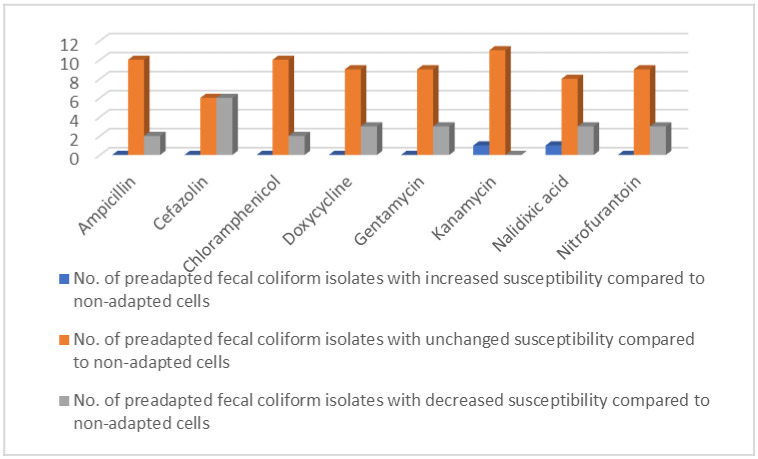
The number of preadapted fecal coliform isolates with changed or unchanged susceptibility to various antibiotics, compared to non-adapted cells, according to the breakpoints. Preadaptation did not change the MICs of ciprofloxacin, streptomycin, and trimethoprim, and these antibiotics are excluded from the figure.

It was observed that the preadaptation process, in the vast majority of cases, either did not change or decreased the susceptibility of the fecal coliforms to the tested antibiotics, changing from resistant/intermediate resistant to intermediate resistant/sensitive (Figure 2).

The increase in MIC only changed the susceptibility of two fecal coliform isolates from sensitive to resistant/intermediate resistant, one to kanamycin and another to nalidixic acid.

## 3. Discussion

In this study, it was observed that the MICs of some of the antibiotics against certain fecal coliform isolates were unchanged or decreased by the preadaptation treatments in two different suboptimal concentrations of ClO_2_ (Table 1 and Figure 1). Similarly, preadaptation either did not change or decreased the susceptibility of some of the fecal coliform isolates to the evaluated antibiotics (Table 2 and Figure 2). Consistent with the results of the present study, Alonso-Hernando et al. [10] reported that the preadaptation of *S. enteritidis* in ClO_2_ did not change its susceptibility to gentamicin, amoxicillin–clavulanic acid, ciprofloxacin, and tetracycline. The pre-exposure of *Salmonella* and *Campylobacter* to ClO_2_ did not change their susceptibility to ampicillin, chloramphenicol, ciprofloxacin, gentamicin, kanamycin, nalidixic acid, streptomycin, tetracycline, and trimethoprim/sulfamethoxazole [11]. The exposure of *E. coli* to sub-inhibitory concentrations of another oxidizing sanitizer, sodium hypochlorite, did not change its susceptibility to several antibiotics, including ampicillin, gentamicin, and ciprofloxacin [12]. Soumet et al. [13] observed that the pre-exposure of *E. coli* to a quaternary ammonium cation led to a four-fold decrease in the MICs of ciprofloxacin, nalidixic acid, trimethoprim, and ampicillin. These phenomena can be explained by the mechanistic variances in the effect of ClO_2_ vs. the evaluated antibiotics on bacterial cells, or by the differences in the location and regulation of the genes encoding bacterial resistance against the two different types of antimicrobial agents [14].

Treatment with a lower ClO_2_ concentration than that recommended by the manufacturer may not kill fecal coliform bacterial cells, but likely exerts sub-lethal injuries to them. Sub-lethal injuries have been reported to cause increased, or even newly developed, sensitivity of damaged cells to selective agents in microbiological media [15,16], as well as antimicrobials or similar substances; for example, sub-lethal treatments have made some bacterial pathogens lose their tolerance to salt, increase their susceptibility to deoxycholate, and become sensitive to sodium azide.

Other than enhanced susceptibility to selective agents used in microbiological media and other antimicrobials, cellular modifications, such as the leakage of intracellular materials and modified metabolic activities, have also been observed in sub-lethally injured bacterial cells [17]. Oxidizing agents, such as ClO_2_, generate hydroxy radicals, which may interfere with the activity of intracellular enzymes and the metabolic pathways in microbial cells, which could lead to decreased susceptibility to antibiotics [18]. ClO_2_ also inhibits the activity of cell membrane proteins, increasing the permeability of the outer and cytoplasmic membranes [19]. Loss of integrity of the cytoplasmic membrane may lead to more effective intake of antibiotics and, eventually, increased susceptibility of bacterial cells to antibiotics [20].

In the current study, preadaptation with sub-lethal concentrations of ClO_2_ increased the MICs of kanamycin against two of the fecal coliform isolates, and the MIC of nalidixic acid against one of the fecal coliform isolates (Table 1 and Figure 1). In a previous study, repeatedly exposing the cells of a poultry isolate of *S. typhimurium* to increasing sub-lethal concentrations of ClO_2_ resulted in a 1.13-fold increase in the MIC of streptomycin compared to unexposed cells [1]. When bacteria cells were pretreated with chlorhexidine, resistance to ceftazidime, sulfamethoxazole, imipenem, cefotaxime, and tetracycline was induced [7,21,22,23]. The mechanism of cross-protection between ClO_2_ and antibiotics has not yet been determined. It has been suggested that the possible linkage between resistance to antibiotics and sanitizers might be due to common resistance mechanisms, such as the involvement of multidrug efflux pumps [15] and the production of stress response proteins [24].

It is worth noting that this study evaluated the in vitro MICs of various antibiotics against selected bacterial isolates. It is not known whether the results have any relevance from a biological or clinical standpoint.

## 4. Materials and Methods

Cells of fecal coliform bacteria (*n* = 12), F5, F8, F35, F252, F219, F112, F272, F174, F329, F354, F390, and F406, previously isolated from fresh produce packing facilities in a separate research project of our laboratory [25], were used in this study. According to the results of 16S rDNA sequencing, they belong to the genus *Klebsiella*, *Enterobacter*, *Pantoea*, or *Raoultella* (Table 3) [26]. The isolates were retrieved from frozen storage and sub-cultured twice on tryptic soy agar (Becton Dickinson, Franklin Lakes, NJ) at 37 °C for 24 h. A single colony of each fecal coliform culture was transferred to 9 mL Mueller–Hinton (MH) broth (Becton Dickinson), and the broth cultures were incubated at 37 °C for 16 h.

A commercial, ClO_2_-generating product, Selectrocide-2L500-(Selective Micro Technologies, Dublin, OH, USA), was used to generate chlorine dioxide in this study. The product arrived in solid form and produced 2 L of 500 ppm ClO_2_ solution when activated by the addition of sterile distilled water. The activated product was diluted on the day of the experiment to a working concentration of 5 or 2.5 ppm with sterile distilled water.

The fecal coliform cultures described above were diluted to 10^5^ CFU/mL using a double-strength MH broth, and each diluted culture was then mixed with an equal volume of the 5 or 2.5 ppm ClO_2_ solution with a vortex, generating a fecal coliform cell suspension in a single-strength MH broth containing 2.5 or 1.25 ppm ClO_2_. The fecal coliform cells were adapted in the MH broth with the suboptimal concentrations of ClO_2_ at room temperature for 1 min before being mixed with an equal volume of Dey–Engley (DE) neutralizing broth (Becton Dickinson). The MICs of the adapted and non-adapted cells of the fecal coliforms were determined using the protocols described below.

To obtain the MICs, adapted and non-adapted cells of each fecal coliform culture in the DE neutralizing broth were centrifuged for 5 min at 4200× *g*. The supernatant was removed, and the cell pellet was rinsed twice with sterile deionized water. After the final wash, 0.5 mL of each bacterial cell suspension in sterile deionized water was added into a series of 4.5 mL of MH broth containing an appropriate concentration (2-fold dilution series from 2 to 256 µg/mL) of each of the 13 antibiotics, including ampicillin, cefazolin, cephalothin, chloramphenicol, ciprofloxacin, doxycycline, gentamicin, kanamycin, nalidixic acid, nitrofurantoin, streptomycin, tetracycline, and trimethoprim (Oxoid Ltd., Basingstoke, Hants, UK). The MH broth with the inoculated fecal coliforms was incubated at 37 °C for 16–20 h. The lowest concentration of an antibiotic that inhibited the growth of the bacterial cells after overnight incubation was regarded as the MIC. The MICs of the antibiotics for the sanitizer-adapted and non-adapted cells of fecal coliforms were compared. Susceptibility categorization was assessed according to the current susceptibility and resistant breakpoints advised by the Clinical and Laboratory Standards Institute [27]. Each experiment was conducted in two independent trials.

## 5. Conclusions

This study investigated the changes in antibiotic resistance of selected fecal coliform bacteria after being preadapted with sub-lethal concentrations of ClO_2_. Most fecal coliform bacteria used in the study demonstrated either the same or reduced resistance to the evaluated antibiotics, but slightly stronger resistance to nalidixic acid and kanamycin was demonstrated by one to two of the fecal coliform isolates. The results suggest that the use of ClO_2_ had a relatively low risk of inducing fecal coliform resistance to antibiotics.

## Figures and Tables

**Table 1:** Minimum inhibitory concentrations of various antibiotics against selected fecal coliforms preadapted in suboptimal concentrations of ClO_2_.

			Minimal Inhibitory Concentrations (µg/mL)											
Fecal Coliform Isolates			F5	F8	F35	F252	F219	F112	F272	F174	F329	F354	F390	F406
Antibiotics	Adaptation	ClO_2_ (ppm)												
Ampicillin	W	2.50	32	16	16	16	16	16	32	16	16	8	32	16
	W	1.25	32	*8*	16	16	16	16	32	16	16	8	32	*8*
	W/O		32	16	16	16	16	16	32	16	16	8	32	16
Cefazolin	W	2.50	*16*	*16*	8	*16*	*16*	32	16	32	64	16	*16*	*16*
	W	1.25	*16*	*16*	8	*16*	*16*	32	16	32	64	16	*16*	*16*
	W/O		32	32	8	64	32	32	16	32	64	16	32	32
Cephalothin	W	2.50	*16*	*16*	*4*	*16*	*16*	32	16	32	*64*	16	*16*	*16*
	W	1.25	*16*	*16*	*4*	*16*	*16*	32	16	32	*64*	16	*64*	*16*
	W/O		128	32	8	128	32	32	16	32	128	16	128	32
Chloramphenicol	W	2.50	*32*	32	8	128	*64*	64	16	64	64	*8*	*32*	32
	W	1.25	*64*	32	8	128	*64*	64	16	64	64	*8*	*64*	32
	W/O		128	32	8	128	128	64	64	128	128	16	128	32
Ciprofloxacin	W	2.50	8	16	16	8	8	16	8	16	16	8	8	16
	W	1.25	8	16	16	8	8	16	8	16	16	8	8	16
	W/O		8	16	16	8	8	16	8	16	16	8	8	16
Doxycycline	W	2.50	16	*8*	16	32	32	*32*	16	*32*	16	*8*	16	16
	W	1.25	16	16	16	*16*	32	*16*	16	*32*	*8*	16	16	16
	W/O		16	16	16	32	32	64	16	64	16	16	16	16
Gentamycin	W	2.50	8	*8*	*8*	8	8	*8*	8	16	8	8	8	8
	W	1.25	8	*8*	*8*	8	8	*8*	8	16	8	8	8	8
	W/O		8	16	16	8	8	16	8	16	8	8	8	8
Kanamycin	W	2.50	8	*8*	8	*8*	8	8	8	*8*	*8*	8	8	8
	W	1.25	8	*8*	16	*8*	8	8	32	*8*	*8*	8	8	8
	W/O		8	16	8	16	8	8	16	16	16	8	8	8
Nalidixic acid	W	2.50	*32*	64	16	*64*	64	32	32	64	128	32	32	64
	W	1.25	64	*8*	32	*64*	64	64	32	64	64	16	64	8
	W/O		64	64	16	128	64	64	64	64	128	32	64	64
Nitrofurantoin	W	2.50	32	*16*	*16*	*16*	*16*	*16*	32	*16*	*16*	*8*	32	*16*
	W	1.25	32	*16*	*16*	*16*	*16*	*16*	32	*16*	*16*	*8*	32	*16*
	W/O		32	32	64	64	32	32	32	32	64	16	32	32
Streptomycin	W	2.50	16	8	16	*8*	*8*	*8*	32	16	16	8	16	8
	W	1.25	16	8	16	*8*	16	*8*	32	16	16	8	16	8
	W/O		16	8	16	16	16	16	32	16	16	8	16	8
Tetracycline	W	2.50	16	*8*	16	32	*32*	*32*	16	*32*	16	*8*	16	*8*
	W	1.25	16	16	16	*16*	*32*	*16*	16	*32*	*8*	16	16	16
	W/O		16	16	16	32	64	64	16	64	16	16	16	16
Trimethoprim	W	2.50	32	16	16	8	8	8	16	16	64	32	32	16
	W	1.25	32	16	16	8	8	8	16	16	64	32	32	16
	W/O		32	16	16	8	8	8	16	16	64	32	32	16

W—with adaptation in suboptimal concentrations of ClO_2_; W/O—without adaptation in suboptimal concentrations of ClO_2_; control samples; increased antibiotic MICs are highlighted in bold; decreased antibiotic MICs are in italics.

**Table 2:** Changes in the susceptibility of selected fecal coliforms to various antibiotics, as affected by preadaptation in sub-lethal concentrations of ClO_2_.

			Susceptibility to Tested Antibiotics											
Fecal Coliform Isolates			F5	F8	F35	F252	F219	F112	F272	F174	F329	F354	F390	F406
Antibiotics (Breakpoints)	Adaptation	ClO_2_ (ppm)												
Ampicillin ≤8, 16, ≥32	W	2.50	R	I	I	I	I	I	R	I	I	S	R	I
	W	1.25	R	*S*	I	I	I	I	R	I	I	S	R	*S*
	W/O		R	I	I	I	I	I	R	I	I	S	R	I
Cefazolin ≤16, -, ≥32	W	2.50	*S*	*S*	S	*S*	*S*	R	S	R	R	S	*S*	*S*
	W	1.25	*S*	*S*	S	*S*	*S*	R	S	R	R	S	*S*	*S*
	W/O		R	R	S	R	R	R	S	R	R	S	R	R
Chloramphenicol ≤8, 16, ≥32	W	2.50	R	R	S	R	R	R	*I*	R	R	*S*	R	R
	W	1.25	R	R	S	R	R	R	*I*	R	R	*S*	R	R
	W/O		R	R	S	R	R	R	R	R	R	I	R	R
Doxycycline ≤4, 8, ≥16	W	2.50	R	*I*	R	R	R	R	R	R	R	*I*	R	R
	W	1.25	R	R	R	R	R	R	R	R	*I*	R	R	R
	W/O		R	R	R	R	R	R	R	R	R	R	R	R
Gentamycin ≤4, 8, ≥16	W	2.50	I	*I*	*I*	I	I	*I*	I	R	I	I	I	I
	W	1.25	I	*I*	*I*	I	I	*I*	I	R	I	I	I	I
	W/O		I	R	R	I	I	R	I	R	I	I	I	I
Kanamycin	W	2.50	S	S	S	S	S	S	S	S	S	S	S	S
≤16, 32, ≥64	W	1.25	S	S	S	S	S	S	*I*	S	S	S	S	S
	W/O		S	S	S	S	S	S	S	S	S	S	S	S
Nalidixic acid ≤16, -, ≥32	W	2.50	R	R	S	R	R	R	R	R	R	R	R	R
	W	1.25	R	S	R	R	R	R	R	R	R	S	R	S
	W/O		R	R	S	R	R	R	R	R	R	R	R	R
Nitrofurantoin ≤32, 64, ≥128	W	2.50	S	S	*S*	*S*	S	S	S	S	*S*	S	S	S
	W	1.25	S	S	*S*	*S*	S	S	S	S	*S*	S	S	S
	W/O		S	S	I	I	S	S	S	S	I	S	S	S
Tetracycline ≤4, 8, ≥16	W	2.50	R	*I*	R	R	R	R	R	R	R	*I*	R	*I*
	W	1.25	R	R	R	R	R	R	R	R	*I*	R	R	R
	W/O		R	R	R	R	R	R	R	R	R	R	R	R

W—with adaptation in suboptimal concentrations of ClO_2_; W/O—without adaptation in suboptimal concentrations of ClO_2_; control samples; increased antibiotic susceptibilities are highlighted in bold; decreased antibiotic susceptibilities are in italics; MIC breakpoints for ciprofloxacin are not available, and the antibiotic is not included in the table; preadaptation did not change the MICs of ciprofloxacin, streptomycin, and trimethoprim, and these antibiotics are excluded from the table; MIC breakpoints for antibiotics were described by the Clinical and Laboratory Standards Institute (CLSI), except for streptomycin. Streptomycin resistance breakpoint was described by the US Food and Drug Administration; S: susceptible; I: intermediate resistant; R: resistant.

**Table 3:** Identities of the bacterial isolates used in the study.

Strain ID	Genus Name
F5	*Klebsiella*
F8	*Pantoea*
F35	*Klebsiella*
F252	*Klebsiella*
F219	*Enterobacter*
F112	*Enterobacter*
F272	*Klebsiella*
F112	*Enterobacter*
F329	*Klebsiella*
F354	*Raoultella*
F390	*Klebsiella*
F406	*Raoultella*

## Data Availability

Data are contained within the article.
